# Trend changes and factor analysis of endometrial hyperplasia in patients with polycystic ovarian syndrome based on the Korean National Health Insurance Database

**DOI:** 10.1186/s12905-022-02015-2

**Published:** 2022-11-08

**Authors:** Bora Park, Hakmo Lee, Suyeon Park, Eun Sil Lee, Jeong Jae Lee, Young Lee, Je Hyun Seo

**Affiliations:** 1 Department of Obstetrics and Gynecology, Soonchunhyang University, College of Medicine, Seoul, South Korea; 2 Veterans Medical Research Institute, Veterans Health Service Medical Center, Jinhwangdo-ro 61-gil 53, Gangdong-gu, Seoul, 05368 South Korea; 3 Department of Biostatistics, Soonchunhyang University, College of Medicine, Seoul, South Korea; 4 Department of Applied Statistics, Chung-Ang University, Seoul, South Korea

**Keywords:** Polycystic ovary syndrome, Endometrial hyperplasia, Korea National Health Insurance claim database, Prevalence, Incidence

## Abstract

**Background:**

Polycystic ovary syndrome (PCOS) is a common endocrine disorder associated with an increased risk of other gynecological disorders, such as endometrial hyperplasia (EH). However, substantial factors in the comorbidity of EH and PCOS remain to be investigated. We analyzed trend changes in PCOS and factors related to the comorbidity of PCOS and EH using data from the Korea National Health Insurance (KNHI) claims database.

**Methods:**

The data for this population-based study of people diagnosed with PCOS or EH in Korea from 2009 to 2016 were collected from the KNHI claims database between 2007 and 2017. We conducted a trend analysis of the prevalence and incidence of PCOS and EH. In addition, we performed a logistic regression analysis to identify risk factors associated with EH incidence in people with PCOS using the matched case-control methodology.

**Results:**

The average annual growth rate of the incidence of PCOS was 14.1% from 2009 to 2016, whereas the EH rate increased by only 3.4% annually. Comorbidities, type 2 diabetes, obesity, hypertension, hyperlipidemia, and infertility, increased the risk of EH in PCOS patients. Additionally, the cumulative duration of oral contraceptive & progestin treatment for PCOS correlated highly with the comorbidity of EH and PCOS.

**Conclusions:**

We confirmed the relationship between PCOS and EH using big data suitable for time series analyses of the diagnosis and treatment of diseases. Endometrial evaluation should be done with more caution if oral contraceptives & progestins have been used for a long time.

**Supplementary Information:**

The online version contains supplementary material available at 10.1186/s12905-022-02015-2.

## Background

Polycystic ovary syndrome (PCOS) is a common endocrine disorder in people of reproductive age that affects 4–18% of that population [1–3]. It is a heterogeneous disorder whose principal features are excess androgen, ovulatory dysfunction, and polycystic ovaries [4]. Insulin resistance and obesity with hyperinsulinemia commonly occur in women with PCOS, and it is associated with hirsutism, chronic anovulation, reduction of average progesterone levels, and infertility [4–10]. In PCOS patients, endometrial proliferation can increase the risk of endometrial hyperplasia (EH) [11].

EH is an increased volume of endometrial tissue with alterations in the glandular architecture that arises in the presence of chronic exposure to estrogen unopposed by progesterone [12]. EH is a pre-cancerous, non-invasive proliferation of the endometrium associated with abnormal menstruation, heavy menstrual bleeding, and infertility [13]. Although the prevalence of EH in people with PCOS varies from 1 to 48.8% [6], few data are available on the exact prevalence of PCOS or EH in the population, especially in Asian countries. Although, several epidemiologic studies have reported the prevalence of PCOS in various populations using different definitions, it ranged from 4 to 21%, depending on the diagnostic criteria [14, 15]. Those variations are caused by limitations in sampling and outcome definitions and could be partly explained by racial differences in the prevalence of hirsutism. In addition, the real-world trend data for EH in people with PCOS are insufficient. Therefore, national-level epidemiologic data are needed to understand the natural course of PCOS and validate its relationships with comorbid disorders. The first goal of this study was to investigate the trend changes in incidence and prevalence of PCOS and EH, respectively, using a nationally representative health insurance claim database. The second goal was to identify factors associated with EH in people with PCOS.

## Methods

### Data source

This nationwide, population-based, retrospective cohort study used Korean Health Insurance Review and Assessment (HIRA) data from January 1, 2007, through September 30, 2017. The HIRA database uses diagnosis codes from the 10th revision of the International Classification of Diseases (ICD-10). South Korea has a national health insurance system that covers all medical services for 97% of the population. Under that system, Korean medical institutions are required to submit most of their medical records services—with the exception of noninsurance procedures such as plastic surgery—to the HIRA database. Medical records for the remaining 3% of the population, who are covered by the National Medical Aid program, are also submitted to HIRA. Thus, the HIRA database contains information about all medical claims, including sex, age, diagnosis, treatments, procedures, and prescription history, for approximately 50 million Koreans [16]. Access to HIRA data is regulated by HIRA rules on data exploration and utilization. We used the data after gaining HIRA approval.

### Study population

**Fig. 1:**
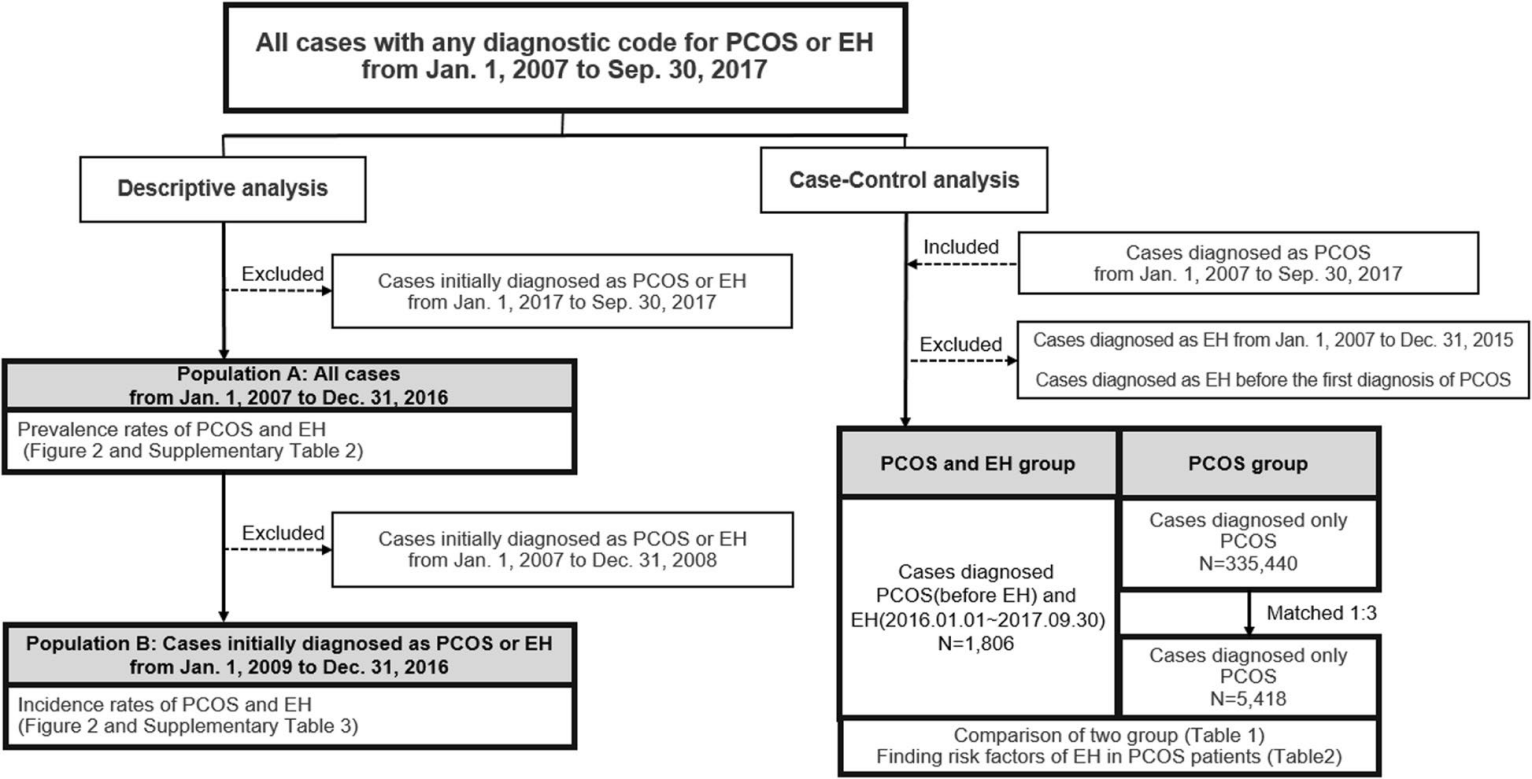
Schematic flow of study design. PCOS = polycystic ovary syndrome, EH = endometrial hyperplasia

We accessed data for everyone in the HIRA database who had been diagnosed with PCOS (ICD-10, E282) or EH (ICD-10, N850, N851) from January 1, 2007, to September 30, 2017. A schematic diagram showing the flow of the study design is provided in Fig. 1. We conducted descriptive analyses to find trend changes and a case-control study to analyze the factors involved in the comorbidity of EH and PCOS. For the case-control study, we defined PCOS patients diagnosed with EH for the first time after 2015 as cases. The PCOS diagnosis date for those cases is thus prior to their EH diagnosis. Follow up duration was calculated from the diagnosis of PCOS to the onset of EH or final follow-up. The age distribution- and follow-up duration-matched controls were patients with PCOS but not EH.

### Outcome of interest for the trend analysis and clinical factors for the case-control analysis

The prevalence rates of PCOS and EH were calculated from 2009 to 2016. We set 2007 and 2008 as a washout period, and the incidence rate of each condition was estimated from 2009 to 2016. We conducted a matched case-control study to identify risk factors for EH in PCOS patients. As risk factors for acquiring EH after a PCOS diagnosis, we considered age, region, type of health care delivery system, comorbidities, and duration of comedications. The comorbidities we considered were type 1 diabetes (T1D), type 2 diabetes (T2D), hypertension (HT), hyperlipemia (HL), obesity, and infertility. T1D was defined as ICD-10 code E10, T2D as E11–E14, HT as E78, infertility as N96 or N97, and obesity as E66. Comedications considered were oral contraceptives & progestin, excess androgen treatments (antiandrogens), and infertility treatments. (Medication codes are shown Supplementary Table 1.) Medication duration was calculated from the diagnosis of PCOS to the onset of EH or final follow-up. Age was categorized as younger than 30 years, 30s, 40s, and 50 years or older. Cumulative duration of comedication was categorized as nonusers, fewer than 180 days, and 180 days or more.

### Statistical analysis

Prevalence and incidence rates were determined using the number of individuals diagnosed with PCOS or EH as the numerator and the number of Korean females in the population for each year based on the KOSIS database (https://kosis.kr/eng/) as the denominator. To better delineate disease growth, we also calculated an annual growth rate (% increase/year) using the geometric mean. Characteristics of PCOS patients (age, medications, region, type of health care delivery system, and comorbidities) were analyzed. We matched patients with and without EH in a 1:3 ratio according to the categorized age distribution and follow-up duration. Propensity score matching was performed with the use of the nearest-neighbor matching algorithm (caliper width, 0.2 of the standard deviation of the logit score). Differences in continuous and categorical variables according to the presence of EH were analyzed using independent t-testing and Pearson’s chi-square test or Fisher’s exact test, respectively. An unconditional logistic regression analysis was used to identify risk factors associated with EH incidence in people with PCOS because we used loose matching data. The cumulative duration of drugs and variables with a *p*-value less than 0.2 in the simple logistic regression were included in the multiple logistic regression model. Variable selection was performed through a backward process based on Akaike information criterion values. Associations between clinical factors and EH were summarized as odds ratios (ORs) and 95% confidence intervals (CIs). All statistical analyses were performed using SAS version 9.3 (SAS Institute Inc., Cary, NC, USA) and R statistical software, version 3.4.1 (R Foundation for Statistical Computing, Vienna, Austria). Statistical significance was established as two-sided *p*-values < 0.05.

## Results

### Trends in the prevalence and incidence rates for PCOS and EH

**Fig. 2:**
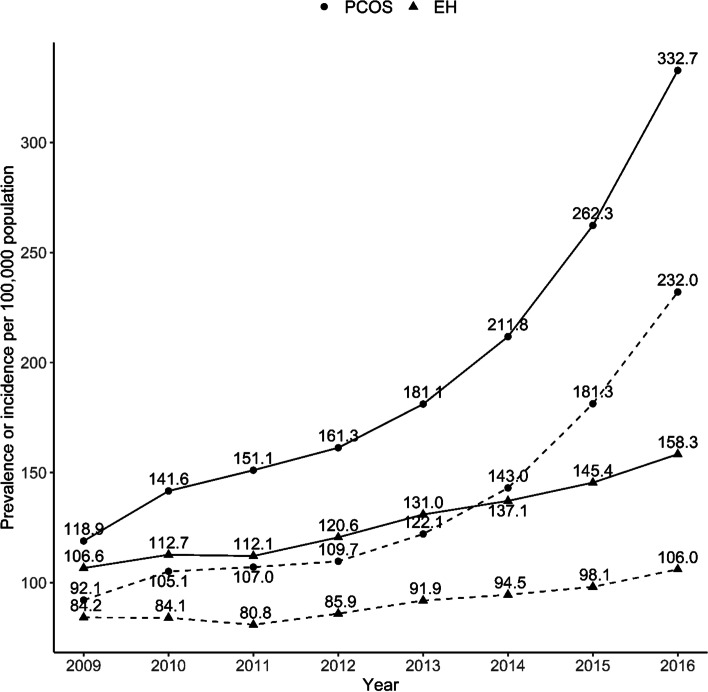
Trend changes in PCOS and EH: Prevalence and incidence rates of PCOS and EH per 100,000 people from 2009 to 2016. Prevalence is indicated as a bold line, and incidence is indicated as a dotted line

The overall PCOS prevalence rates from 2009 to 2016 were 118.9, 141.6, 151.1, 161.3, 181.1, 211.8, 262.3, and 332.7 per 100,000 people, respectively, with an average annual growth rate (AAGR) of 15.8%. The prevalence rates for EH during that period ranged from 106.6 to 158.3 per 100,000 people, and the AAGR was 5.8 (Fig. 2 and Supplementary Table 2). The total crude rates (CRs) for overall PCOS incidence from 2009 to 2016 were 92.1, 105.1, 107, 109.7, 122.1, 143, 181.3, and 232 per 100,000 people, respectively. The CRs for EH during that period ranged from 80.8 to 106 per 100,000 people. The AAGR of PCOS incidence was 14.1% (17.4% among those younger than 30, 10.9% among those in their 30s, 26.8% among those in their 40s, and 33.7% among those 50 years or older) from 2009 to 2016 (Supplementary Table 3). A rapid increase in PCOS incidence was seen between 2012 and 2016. In contrast, the incidence of EH increased only slightly, by 3.4% annually (Fig. 2).

### Trend changes by age distribution and the class of PCOS medications

**Fig. 3:**
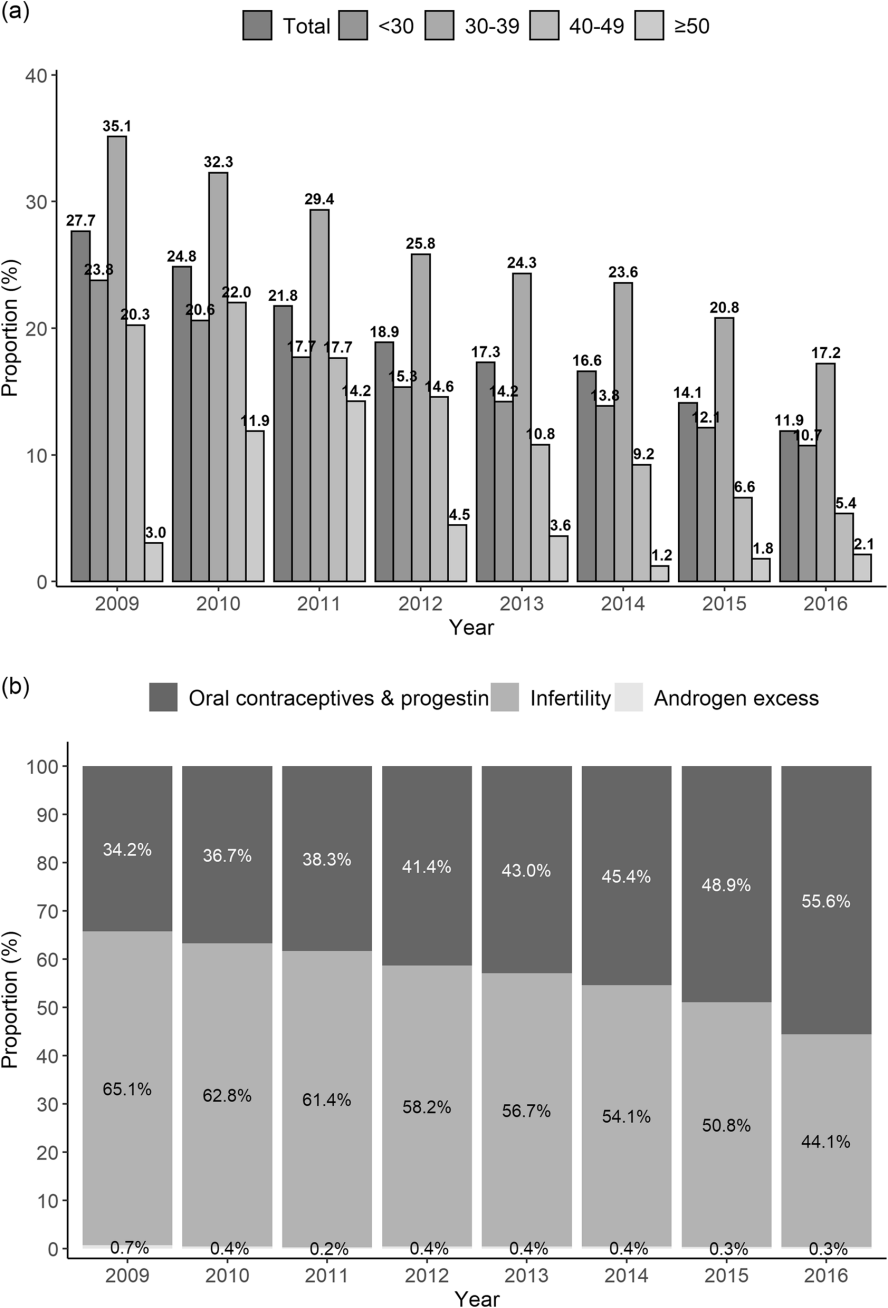
Trend changes by age distribution and class of PCOS medication. **a** Proportion of patients given medications for PCOS per hospital visit by age distribution. **b** Annual trends in PCOS-related prescription medications: oral contraceptives & progestin, infertility, and excess androgen (antiandrogen)

From 2009 to 2016, 69,189 patients who visited the hospital received a PCOS diagnosis and were given medications at the same time. The proportion of PCOS patients who received prescriptions for disease-related drugs from 2009 to 2016 was 27.7, 24.8, 21.8, 18.9, 17.3, 16.6, 14.1, and 11.9%, respectively. The rates among those younger than 30 years were highest among the age groups, ranging from 10.7 to 23.8%, whereas those among patients 50 years or older ranged were the lowest, from 1.2 to 14.2% (Fig. 3 a). Looking at yearly trends in PCOS-related prescription drugs, the rate of prescriptions for oral contraceptives & progestin increased to 21.4% between 2009 and 2016, whereas medications related to infertility decreased to 21% in the same period (Fig. 3 b). Medications related to excess androgen did not change significantly, decreasing from 0.7 to 0.3% during 7 years.

### Characteristics of enrolled PCOS patients according to the presence of EH

For the case-control analysis, we studied 1806 people with PCOS who were diagnosed with EH as a comorbid condition and 335,440 people who had only PCOS. The mean age of the patients with EH was older than that of the people without EH (35.3 ± 8.0 years vs. 31.4 ± 8.3 years, *P* < 0.001, Table 1). The only factors we examined that did not differ significantly between the groups were antiandrogen use (*P* = 0.776) and the proportion of T1D patients (*P* = 0.221). The use of oral contraceptives & progestin and infertility medications was significantly higher in the EH group (P < 0.001). Among the comorbidities, T2D, obesity, HT, HL, and infertility were all significantly higher in the EH group (all *P* < 0.001). The follow-up duration was approximately 200 days longer in patients without EH (P < 0.001). After matching by age distribution and follow-up duration, the absolute standardized difference in age and follow-up duration between people with and without EH was less than 0.1, which was a negligible difference [17]. Even after matching, the use of oral contraceptives & progestin and infertility medications and the comorbidities of T2D, obesity, HT, HL, and infertility were significantly higher in the EH group (all *P* < 0.001).

**Table 1:** Characteristics of enrolled PCOS patients according to the presence of EH

	Before matching			After matching			
	With EH	Without EH	***p*** value	With EH	Without EH	***p*** value^**a**^	Standardized
	**(*n*** **= 1806)**	**(*n*** **= 335,440)**		(n= 1806)	(n= 5418)		**difference**^**b**^
Age, years	35.3 ± 8.0	31.4 ± 8.3	< 0.001	35.3 ± 8.0	34.8 ± 8.1	0.007	0.062
Age distribution			< 0.001			0.715	0.039
< 20 yr	14 (0.8)	15,989 (4.8)		14 (0.8)	47 (0.9)		
20–29 yr	414 (22.9)	135,556 (40.4)		414 (22.9)	1288 (23.8)		
30–39 yr	868 (48.1)	132,156 (39.4)		868 (48.1)	2578 (47.6)		
40–49 yr	423 (23.4)	42,340 (12.6)		423 (23.4)	1281 (23.6)		
≥50 yr	87 (4.8)	9399 (2.8)		87 (4.8)	224 (4.1)		
Infertility medications	565 (31.3)	74,512 (22.2)	< 0.001	565 (31.3)	1434 (26.5)	< 0.001	0.106
Oral contraceptives & progestin	761 (42.1)	95,839 (28.6)	< 0.001	761 (42.1)	1364 (25.2)	< 0.001	0.364
Excess androgen medication	23 (1.3)	3936 (1.2)	0.776	23 (1.3)	65 (1.2)	0.901	0.009
Cumulative duration of infertility medication use, days	6.4 ± 14.9	3.9 ± 14.0	< 0.001	6.4 ± 14.9	4.5 ± 13.1	< 0.001	0.135
Cumulative duration of oral contraceptive use, days	13.6 ± 51.1	7.7 ± 51.3	< 0.001	13.6 ± 51.1	6.2 ± 30.8	< 0.001	0.175
Cumulative duration of antiandrogen medication, days	0.7 ± 9.9	1.3 ± 38.0	0.005	0.7 ± 9.9	0.6 ± 11.4	0.829	0.009
Cumulative duration of infertility medication			< 0.001			< 0.001	0.107
Nonuser	1241 (68.7)	260,928 (77.8)		1241 (68.7)	3984 (73.5)		
< 180 days	564 (31.2)	74,454 (22.2)		564 (31.2)	1431 (26.4)		
≥ 180 days	1 (0.1)	58 (0.0)		1 (0.1)	3 (0.1)		
Cumulative duration of oral contraceptives & progestin			< 0.001			< 0.001	0.366
Nonuser	1045 (57.9)	239,601 (71.4)		1045 (57.9)	4054 (74.8)		
< 180 days	743 (41.1)	94,153 (28.1)		743 (41.1)	1341 (24.8)		
≥ 180 days	18 (1.0)	1686 (0.5)		18 (1.0)	23 (0.4)		
Cumulative duration of antiandrogen medication			0.770			0.940	0.007
Nonuser	1783 (98.7)	331,504 (98.8)		1783 (98.7)	5353 (98.8)		
< 180 days	21 (1.2)	3399 (1.0)		21 (1.2)	59 (1.1)		
≥ 180 days	2 (0.1)	537 (0.2)		2 (0.1)	6 (0.1)		
Region			0.008			0.002	0.084
Non-metropolitan region	872 (48.3)	151,463 (45.2)		872 (48.3)	2388 (44.1)		
Metropolitan region	934 (51.7)	183,977 (54.8)		934 (51.7)	3030 (55.9)		
Type of health care delivery system			< 0.001			< 0.001	0.418
Tertiary care	195 (10.8)	15,998 (4.8)		195 (10.8)	283 (5.2)		
Secondary care	621 (34.4)	66,274 (19.8)		621 (34.4)	1112 (20.5)		
Primary care	990 (54.8)	253,168 (75.5)		990 (54.8)	4023 (74.3)		
Type I diabetes	19 (1.1)	2587 (0.8)	0.221	19 (1.1)	39 (0.7)	0.223	0.042
Type II diabetes	355 (19.7)	44,773 (13.3)	< 0.001	355 (19.7)	679 (12.5)	< 0.001	0.197
Obesity	72 (4.0)	7492 (2.2)	< 0.001	72 (4.0)	89 (1.6)	< 0.001	0.146
Hypertension	198 (11.0)	21,531 (6.4)	< 0.001	198 (11.0)	348 (6.4)	< 0.001	0.164
Hyperlipidemia	816 (45.2)	115,036 (34.3)	< 0.001	816 (45.2)	1742 (32.2)	< 0.001	0.269
Infertility	713 (39.5)	88,022 (26.2)	< 0.001	713 (39.5)	1747 (32.2)	< 0.001	0.153
Follow up duration, days	3458.1 ± 468.9	3661.9 ± 469.7	< 0.001	3458.1 ± 468.9	3466.0 ± 482.5	0.541	−0.017

Data are presented as mean ± standard deviation or number (%)

*PCOS* Polycystic ovary syndrome, *EH* Endometrial hyperplasia

^a^*P* value was estimated using the χ2 test or Fisher’s exact test for categorical variables and t-testing for continuous variables

^b^*Absolute* standardized difference < 0.1 indicates a negligible difference

### Risks factors for EH in PCOS patients

In the simple logistic regression results, the cumulative durations of infertility medication (< 180 days: OR 1.27 [95% CI 1.13–1.42]) and oral contraceptive & progestin use (< 180 days: OR 2.15 [95% CI 1.92–2.41]; ≥ 180 days: OR 3.04 [95% CI 1.63–5.65]) were associated with an increased risk of EH when compared to nonuser (all *P* < 0.001) (Table 2). The OR for EH was less than 1 when the region was in a metropolitan area than non-metropolitan (*P* = 0.002) or the type of health care delivery system (*P* = 0.047 for secondary care and P < 0.001 for primary care, compared to tertiary care) was small. PCOS patients with T2D (OR 1.71 [95% CI 1.48–1.97]), obesity (OR 1.79 [95% CI 1.49–2.15]), HT (OR 1.74 [95% CI 1.56–1.94]), HL (OR 2.49 [95% CI 1.81–3.41]), or infertility (OR 1.37 [95% CI 1.23–1.53]) showed increased risks of EH (all P < 0.001). The age distribution (all *P* > 0.05), cumulative duration of antiandrogen use (*P* = 0.795 for < 180 days and *P* = 0.999 for ≥180 days, compared to nonuser), T1D (*P* = 0.173), and follow-up duration (*P* = 0.541) did not differ significantly in the univariate analysis. The cumulative duration of drug use, region, type of health care delivery system, and comorbidities were included in the multiple logistic regression model. After variable selection, the cumulative duration of infertility and antiandrogen medication use, and T1D were excluded from the final model. The ORs for oral contraceptive & progestin use (< 180 days = 2.01, 95% CI: 1.79–2.26, *p* < 0.001 and ≥ 180 days = 2.07, 95% CI: 1.09–3.95, *p* = 0.026) increased with the duration of treatment compared with the no medication group. Among comorbidities, T2D, obesity, HT, HL, and infertility all increased the risk of EH (OR = 1.17 [95% CI 1.00–1.38, *P* = 0.050], OR = 1.34 [95% CI: 1.10–1.64, *P* = 0.004], OR = 1.49 [95% CI: 1.31–1.68, *P* < 0.001], OR = 2.17 [95% CI: 1.55–3.02, P < 0.001], and OR = 1.24 [95% CI: 1.10–1.39, P < 0.001], respectively). The association between the type of health care delivery system and EH remained significant in the final model (Table 2).

**Table 2:** Effect of risk factors on the development of EH in PCOS patients

	Simple logistic regression		Multiple logistic regression without variable selection		Multiple logistic regression with variable selection	
	OR (95% CI)	*p* value	OR (95% CI)	*p* value	OR (95% CI)	*p* value
Age distribution						
< 20 yr	reference					
20–29 yr	1.08 (0.59–1.98)	0.806				
30–39 yr	1.13 (0.62–2.06)	0.690				
40–49 yr	1.11 (0.60–2.03)	0.739				
≥ 50 yr	1.30 (0.68–2.49)	0.421				
Cumulative duration of infertility medication						
Nonuser	reference		reference			
< 180 days	1.27 (1.13–1.42)	< 0.001	0.89 (0.74–1.06)	0.192		
≥ 180 days	1.07 (0.11–10.3)	0.953	0.55 (0.05–5.74)	0.616		
Cumulative duration of oral contraceptives & progestin						
Nonuser	reference		reference		reference	
< 180 days	2.15 (1.92–2.41)	< 0.001	2.03 (1.81–2.28)	0.000	2.01 (1.79–2.26)	< 0.001
≥ 180 days	3.04 (1.63–5.65)	< 0.001	2.12 (1.11–4.04)	0.023	2.07 (1.09–3.95)	0.026
Cumulative duration of antiandrogen medication						
Nonuser	reference		reference			
< 180 days	1.07 (0.65–1.76)	0.795	0.76 (0.45–1.28)	0.299		
≥ 180 days	1.00 (0.20–4.96)	0.999	0.69 (0.14–3.54)	0.658		
Region						
Non-metropolitan region	reference		reference		reference	
Metropolitan region	0.84 (0.76–0.94)	0.002	0.92 (0.82–1.02)	0.123	0.92 (0.82–1.03)	0.128
Type of health care delivery system						
Tertiary care	reference		reference		reference	
Secondary care	0.81 (0.66–1.00)	0.047	0.84 (0.68–1.04)	0.118	0.84 (0.68–1.04)	0.114
Primary care	0.36 (0.29–0.43)	< 0.001	0.39 (0.31–0.47)	0.000	0.39 (0.32–0.47)	< 0.001
Type I diabetes	1.47 (0.85–2.54)	0.173	0.79 (0.44–1.44)	0.442		
Type II diabetes	1.71 (1.48–1.97)	< 0.001	1.19 (1.01–1.40)	0.036	1.17 (1.00–1.38)	0.050
Obesity	1.79 (1.49–2.15)	< 0.001	1.35 (1.11–1.66)	0.003	1.34 (1.10–1.64)	0.004
Hypertension	1.74 (1.56–1.94)	< 0.001	1.49 (1.32–1.68)	0.000	1.49 (1.31–1.68)	< 0.001
Hyperlipidemia	2.49 (1.81–3.41)	< 0.001	2.18 (1.57–3.05)	0.000	2.17 (1.55–3.02)	< 0.001
Infertility	1.37 (1.23–1.53)	< 0.001	1.35 (1.14–1.60)	0.001	1.24 (1.10–1.39)	< 0.001
Follow up duration	1.00 (1.00–1.00)	0.541				

*PCOS* Polycystic ovary syndrome, *EH* Endometrial hyperplasia, *OR* Odds ratio, *CI* Confidence interval

## Discussion

In this study, we used big data from a national health insurance database to find trend changes in PCOS and its relationship with EH. Due to the peculiarities of the Korean medical system, big data suitable for use in time-series analyses of disease diagnosis and treatment are available through a unified system, and many studies using those data are being published.

According to our study, the prevalence rates of PCOS in all participants, those younger than 30 years, and those older than 30-years in 2016 were 332.7, 458.4, and 400.4 per 100,00 people, respectively. These are all less than 1%, which is lower than the percentages reported in other studies. The prevalence (estimated) of PCOS among Korean women was 5.8% according to a 2011 study including about 8000 women of reproductive age [18]. According to a Chinese study that analyzed data from medical examination centers, the prevalence rate of PCOS is 2.2% [19]. Estimates of prevalence from community-based or hospital-based studies are likely to be overestimated in comparison to those based on national data. The national PCOS prevalence in Turkey is 258.5 per 100,000 (0.26%), which is lower than the 6.1% estimated by community-based study [20, 21]. The average national PCOS prevalence in Europe was 276.4 per 100,000 people (0.28%) in 2016, which was lower than our results [20]. We estimated the prevalence of EH in 2016 to be 158.3 per 100,000 people. The prevalence of EH in premenopausal women with abnormal uterine bleeding in Japan is 6.2%, which is higher than the prevalence reported in our study (< 0.2%) and that in a Chinese study of infertile women (3.0%) [22, 23]. The prevalence rate varies depending on race, time of year, and participants.

PCOS has a variety of phenotypes, and the diagnostic criteria are controversial. The National Institute of Health (NIH), the European Society of Human Reproduction and Embryology (ESHRE), and the Androgen Excess Society (AES) presented diagnostic criteria for PCOS in 1990, 2003, and 2006, respectively. These variable diagnostic criteria have an impact on PCOS prevalence. The prevalence of PCOS in a community sample of the Iranian population was 7.1% using the NIH, 11.7% according to the AES, and 14.6% according to the ESHRE criteria [24]. In a study conducted in a government-based laboratory with the largest number of female staff (*n* = 527) employed by a single laboratory in Ankara, Turkey, the prevalence of PCOS according to NIH, AES, and ESHRE criteria was 6.1, 15.3, and 19.9%, respectively [21]. In a Chinese meta-analysis, the prevalence of PCOS was high in the order of those aged between 21 and 30, 10 to 20, 31 to 40, and above 40 years (17.23, 10.26, 9.13, and 2.22%, respectively). Furthermore, the majority of PCOS research included only people younger than 50 years [25].

We found that when the incidence of PCOS increased sharply, a non-significant increase occurred in the incidence of EH. EH was thought to occur at a relatively constant rate in people diagnosed with PCOS, but our data did not confirm the expected increase in EH when PCOS diagnoses increased rapidly. A rapid increase in PCOS incidence was seen between 2012 and 2016, but the incidence of EH in those years increased only slightly, by 3.4% annually. In 2009 and 2016, the incidence rates (CRs) of PCOS among people in their 30s were 193.9 and 400.4 per 100,000, respectively. That is relatively high compared with the 71.15 and 73.88 cases per 100,000 of PCOS reported among people of reproductive age in East Asia in 2007 and 2017 [26].

When we examined trend changes by age distribution, we found that those younger than 30 had the highest rate, and those older than 50 had the lowest rate. Those results indicate that PCOS tends to be diagnosed and persist mainly in people in their 20s and 30s.

We found that between 2009 and 2016, 15 to 30% of patients diagnosed with PCOS received medication to treat the disease, with the highest rate of medication usage reported in people younger than 30 years. Prescription rates for medications that offer endometrial protection increased by 21.4% between 2009 and 2016, whereas those for medications to treat infertility decreased by 21%. Medications to treat excess androgen did not change much, from 0.7 to 0.3% over 7 years. Whereas oral contraceptives play a role in controlling ovulatory amenorrhea, which affects the occurrence of EH, medications for infertility can be used to treat hyperandrogenism.

Our factor analysis examining EH in PCOS patients produced some interesting findings. Previous studies reported a positive association between PCOS and EH, on the one hand, and T1D and T2D, on the other, after adjusting for BMI [27–34]. That association has been reported to be specific to obese women [35–37], and excessive insulin circulating through that mechanism can stimulate hypertrophy of endometrial cells, causing EH [38, 39]. A history of HT has also been suggested as a risk factor for EH in several case-control and cohort studies [30, 40–45]. Similar results were found in our study, with T2D, obesity, HT, HL, and infertility increasing the risk of EH in people who already have PCOS. Additionally, the cumulative duration of oral contraceptive & progestin use for PCOS correlated highly with the development of EH.

A major strength of our study is the inclusion of nationwide population data, which provided evidence of an increase in PCOS prevalence and incidence. Additionally, we found several factors that correlate with the presence of EH in people with PCOS. Nonetheless, this study has a few limitations. First, many people in their 40s and 50s with high disease rates were included in the later years of our study period because we built our dataset by extracting data from people with PCOS and EH disease codes, not from all Korean females. Additionally, our data did not allow us to determine whether PCOS is a risk factor for EH. Second, the follow-up period for the PCOS patient group diagnosed later during the data period was not long enough to adequately observe the progression of the disease. When analyzing the factors influencing the occurrence of EH in PCOS patients, only those diagnosed with PCOS within about 10 years prior to the first occurrence of EH were considered. We did not determine the duration from first PCOS diagnosis to first EH diagnosis. Third, medical practices and behaviors not included in the data might have affected the trend changes we found for PCOS and EH. In addition, we did not fully control for confounding effects because of non-measurable potential confounding factors such as laboratory data or patient-reported outcomes. Fourth, databases containing diagnosis codes for diseases might not record information on the severity of the disease, and changes in diagnostic criteria over time might not be accurately distinguished. In particular, PCOS is a heterogeneous disorder that was mapped to a single ICD code despite its various diagnosis criteria. Fifth, since the disease is defined even if there is only one ICD-10 diagnostic code which may be given for the purpose of examination, the disease classification can be ambiguous. There is no indication as to the outcomes following diagnostic labelling. We considered endometrial hyperplasia and endometrial adenoma hyperplasia as endometrial hyperplasia regardless of severity. Sixth, while we evaluated PCOS treatment based on the prescribed drug code following diagnosis of PCOS, we could not rule out that the drug was treatment of other diseases. Therefore, the results must be interpreted with caution, and more accumulated data are needed to further analyze the relationship between PCOS and EH.

## Conclusions

Our trend change analysis showed that both the prevalence and incidence of PCOS increased much more than those of EH during the study period. Of the comorbidities we examined, T2D, obesity, HT, HL, and infertility all increased the risk of EH in people with PCOS. Additionally, the cumulative duration of oral contraceptive & progestin use for PCOS correlated highly with the development of EH. Therefore, endometrial evaluation should be done with more caution if oral contraceptives & progestins have been used for a long time. More large, well-designed studies or pooled analyses could help to clarify the comorbidity of EH and PCOS.

## Supplementary Information

**Additional file 1: Supplementary Table 1.** List of medications.** Supplementary Table 2.** Prevalence rates of PCOS and EH from 2009 to 2016 by age distribution. PCOS = polycystic ovary syndrome, EH = endometrial hyperplasia. **Supplementary Table 3.** Incidence rates of PCOS and EH from 2009 to 2016 by age distribution. PCOS = polycystic ovary syndrome, EH = endometrial hyperplasia.

## Data Availability

The data that support the findings of this study are available from Korean Health Insurance Review and Assessment (HIRA) but restrictions apply to the availability of these data, which were used under license for the current study, and so are not publicly available. Data are however available from the authors upon reasonable request and with permission of HIRA.

## Abbreviations

PCOS: Polycystic ovary syndrome
EH: Endometrial hyperplasia
KNHI: Korea National Health Insurance
HIRA: Korean Health Insurance Review and Assessment
ICD-10: 10th revision of the International Classification of Diseases
T1D: Type 1 diabetes
T2D: Type 2 diabetes
HT: Hypertension
HL: Hyperlipemia
ORs: Odds ratios
CIs: Confidence intervals
AAGR: Average annual growth rate
CRs: Crude rates
