# Supplementary material for: Trend changes and factor analysis of endometrial hyperplasia in patients with polycystic ovarian syndrome based on the Korean National Health Insurance Database
Source: BMC Womens Health. 2022 Nov 8;22:439. doi: 10.1186/s12905-022-02015-2 (PMC9644593; doi:10.1186/s12905-022-02015-2)
Supplement: Supplementary file 1 — Additional file 1: Supplementary Table 1. List of medications. Supplementary Table 2. Prevalence rates of PCOS and EH from 2009 to 2016 by age distribution. PCOS = polycystic ovary syndrome, EH = endometrial hyperplasia. Supplementary Table 3. Incidence rates of PCOS and EH from 2009 to 2016 by age distribution. PCOS = polycystic ovary syndrome, EH = endometrial hyperplasia. [file 12905_2022_2015_MOESM1_ESM.docx]

**Supplementary Table 1.** List of medications

| **Class** | **Medication code** | **Ingredient name** |
| --- | --- | --- |
| Anti-androgen | 139401ATB | cyproterone acetate |
|  | 159001ATB | finasteride |
|  | 231101ATB | spironolactone |
|  | 231102ATB | spironolactone |
|  | 262700ATB | spironolactone |
| Infertility | 109001ATB | anastrozole |
|  | 136201ATB | clomiphene |
|  | 162702BIJ | gonadotropin |
|  | 162730BIJ | gonadotropin |
|  | 162731BIJ | gonadotropin |
|  | 162732BIJ | gonadotropin |
|  | 162803BIJ | gonadotropin |
|  | 162805BIJ | gonadotropin |
|  | 162830BIJ | gonadotropin |
|  | 167202BIJ | GnRH agonist |
|  | 169402BIJ | gonadotropin |
|  | 169404BIJ | gonadotropin |
|  | 182201ATB | letrozole |
|  | 182602BIJ | GnRH agonist |
|  | 182604BIJ | GnRH agonist |
|  | 182630BIJ | GnRH agonist |
|  | 190201BIJ | gonadotropin |
|  | 190202BIJ | gonadotropin |
|  | 244902BIJ | GnRH agonist |
|  | 244930BIJ | GnRH agonist |
|  | 467501BIJ | GnRH agonist |
| Oral contraceptives & progestin | 557100ATB | ethinyl estradiol/ drospirenone |
|  | 557000ATB | ethinyl estradiol/ drospirenone |
|  | 618600ATB | estradiol valerate/dienogest |
|  | 188903ATB | medroxyprogesterone acetate |
|  | 188905ATB | medroxyprogesterone acetate |
|  | 188906ATB | medroxyprogesterone acetate |

**Supplementary Table 2. Prevalence rates of PCOS and EH from 2009 to 2016 by age distribution**

|  | **2009** | **2010** | **2011** | **2012** | **2013** | **2014** | **2015** | **2016** | **Annual growth rate** |
| --- | --- | --- | --- | --- | --- | --- | --- | --- | --- |
| **PCOS, per 100,000 people** | | | | | | | | | |
| **Total** | 118.9 | 141.6 | 151.1 | 161.3 | 181.1 | 211.8 | 262.3 | 332.7 | 15.8 |
| **< 30** | 188.9 | 236.1 | 264.3 | 287.8 | 332.1 | 401.2 | 498.6 | 647.2 | 19.2 |
| **30s** | 262.1 | 312.2 | 327.2 | 352.2 | 387.6 | 448.4 | 527.3 | 642.6 | 13.7 |
| **40s** | 31.8 | 32.5 | 33.1 | 39.5 | 52.5 | 65.8 | 118.6 | 171 | 27.2 |
| **≥ 50** | 2.3 | 2.3 | 2.9 | 3 | 4.8 | 5.5 | 12.8 | 19.3 | 35.8 |
| **EH, per 100,000 people** | | | | | | | | | |
| **Total** | 106.6 | 112.7 | 112.1 | 120.6 | 131 | 137.1 | 145.4 | 158.3 | 5.8 |
| **< 30** | 30 | 28.8 | 27 | 27 | 30.1 | 33 | 35.5 | 39.9 | 4.1 |
| **30s** | 126.5 | 130.4 | 128.5 | 142.1 | 160.7 | 166.3 | 174.8 | 189.8 | 6 |
| **40s** | 268.4 | 285.1 | 284.7 | 304.4 | 328 | 341.6 | 362.3 | 398 | 5.8 |
| **≥ 50** | 95.7 | 104.1 | 104.7 | 111.8 | 117.2 | 121.7 | 129.2 | 138.4 | 5.4 |

PCOS=polycystic ovary syndrome, EH=endometrial hyperplasia

**Supplementary Table 3. Incidence rates of PCOS and EH from 2009 to 2016 by age distribution**

|  | **2009** | **2010** | **2011** | **2012** | **2013** | **2014** | **2015** | **2016** | **Annual growth rate** |
| --- | --- | --- | --- | --- | --- | --- | --- | --- | --- |
| **PCOS, per 100,000 people** | | | | | | | | | |
| **Total** | 92.1 | 105.1 | 107 | 109.7 | 122.1 | 143 | 181.3 | 232 | 14.1 |
| **< 30** | 149 | 181.3 | 193.8 | 201.6 | 230.3 | 278.9 | 349 | 458.4 | 17.4 |
| **30s** | 193.9 | 217.6 | 216.3 | 224.5 | 240.9 | 277.8 | 331.1 | 400.4 | 10.9 |
| **40s** | 26.7 | 24.3 | 24.2 | 28.4 | 39.3 | 49.3 | 97.3 | 140.9 | 26.8 |
| **≥ 50** | 2.1 | 1.9 | 1.9 | 2.4 | 4.3 | 4.8 | 11.4 | 16.2 | 33.7 |
| **EH, per 100,000 people** | | | | | | | | | |
| **Total** | 84.2 | 84.1 | 80.8 | 85.9 | 91.9 | 94.5 | 98.1 | 106 | 3.4 |
| **< 30** | 25.8 | 23.8 | 21.8 | 21.9 | 24.6 | 26.2 | 28.3 | 31.3 | 2.8 |
| **30s** | 102.9 | 102.9 | 97.3 | 108.2 | 122.5 | 122.6 | 126.9 | 136.2 | 4.1 |
| **40s** | 208.4 | 209.7 | 204.2 | 214 | 226.8 | 231.6 | 241.4 | 264 | 3.4 |
| **≥ 50** | 73.4 | 73.7 | 71 | 75.1 | 76.1 | 79.1 | 81 | 86.4 | 2.4 |

PCOS=polycystic ovary syndrome, EH=endometrial hyperplasia
